# Imaging Features of Non-Alcoholic Fatty Liver Disease in Children and Adolescents

**DOI:** 10.3390/children4080073

**Published:** 2017-08-11

**Authors:** Michele Di Martino, Kameliya Koryukova, Mario Bezzi, Carlo Catalano

**Affiliations:** Department of Radiological, Oncological and Pathological Sciences, Sapienza University of Rome, V.le Regina Elena 324 00161 Rome, Italy; kam07@libero.it (K.K.); mario.bezzi@uniroma1.it (M.B.); carlo.catalano@uniroma1.it (C.C.)

**Keywords:** NAFLD, ultrasound, proton density magnetic resonance, magnetic resonance spectroscopy

## Abstract

Non-invasive diagnosis and quantification of liver steatosis is important to overcome limits of liver biopsy, in order to follow up patients during their therapy and to establish a reference standard that can be used in clinical trials and longitudinal studies. Imaging offers several methods in this setting: ultrasound, which is the cheapest technique and easy to perform; magnetic resonance spectroscopy (MRS), which reflects the real content of triglycerides in a specific volume; and proton density fat fraction (PDFF) magnetic resonance, which is a simple method that reflects the distribution of the fat in the whole liver. Other techniques include ultrasound elastography (EUS) and magnetic resonance elastrography (MRE), which can evaluate the progression of non-alcoholic fatty liver disease (NAFLD) into non-alcoholic steato-hepatitis (NASH) and cirrhosis, by quantifying liver fibrosis.

## 1. Introduction

Liver biopsy still represents the reference standard for the diagnosis of liver steatosis in adolescents and children, and corresponds to the mainstay in clinical trials in the enrolment of patients and evaluation of their outcomes. It has some limits such as sampling variability, related to the heterogeneity of the distribution of the steatosis; moreover, in the differentiation between simple steatosis and steato-hepatitis (NASH), complications may also occur (bleeding or infection) and it is not appropriate for screening, longitudinal monitoring, or evaluating treatment response, especially in young population [1,2]. Furthermore, there are inter- and intra-individual variations regarding the degree of steatosis and fat content in the liver that is frequently overestimated when graded semi-quantitatively [3]. In 2012, however, the European Society for Paediatric Gastroenterology Hepatology and Nutrition (ESPGHAN) panel assessed that liver biopsy should not be used as a screening procedure in patients with non-alcoholic fatty liver disease (NAFLD), and its indication can only be based on expert opinions, that must take into consideration a differential diagnosis and the risk of progression of liver disease to cirrhosis. Additionally, non-invasive biochemical and metabolic tests must also be completed [4]. Non-invasive methods for the evaluation of fatty liver in children are crucial, because their disease must be monitored for an extensive period [5]. In this setting, imaging offers several methods that can provide non-invasive analyses of liver steatosis.

## 2. Ultrasound

**Figure 1:**
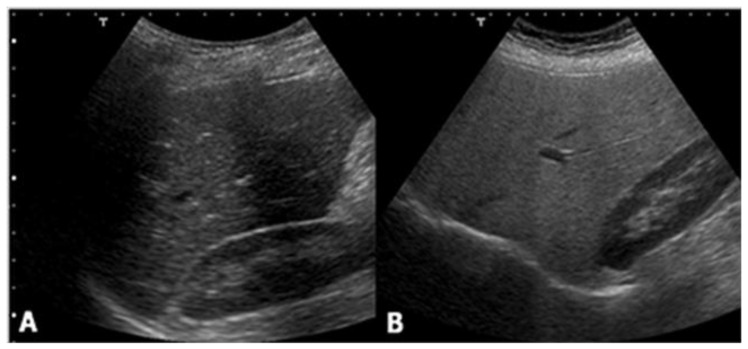
Ultrasound shows no difference between (**A**) the liver and right kidney cortex echogenicity, while (**B**) it demonstrates a hyperechoic liver compared with the kidney parenchyma.

Ultrasound is the most common imaging technique used for the evaluation of liver steatosis. Due to its significant advantages, such as being largely available, relatively non-expensive, and easy to use, ultrasound has been adopted as the standard care for the screening of a patient suspected of having NAFLD. Healthy liver parenchyma shows homogeneous echo texture and similar echogenicity compared to the right kidney cortex. However, in hepatic steatosis, the presence of lipid droplets within hepatocytes disturbs the propagation of the sound wave, causing scatter and attenuation. As waves scatter, more echoes return to the ultrasound transducer. This increase in signal from the liver produces a brighter visualization of liver parenchyma, compared to the kidney. Attenuation of the ultrasound waves also causes depth-dependent loss of signal, leading to an obscuration of vessels and bile ducts, and blurring of the diaphragm (Figure 1).

Classification of liver steatosis through the use of an ultrasound is based on a 4-grade scale:

1. normal (liver echogenicity similar to kidney);
2. mild steatosis (diffuse increase in liver echogenicity);
3. moderate (liver echogenicity obscures vessel walls and the diaphragm)
4. severe (non-visualization of the hepatic vessels and diaphragm).

However, there are two main limits for this technique: an operator and machine dependency and the lack of an objective quantitative analysis [6,7,8]. Moreover, ultrasound has low sensitivity in the differentiation between a healthy liver and mild steatosis (hepatic fat fraction < 30%) [4] and other pathological conditions, such as fibrosis and/or inflammation which may increase liver echogenicity, can sometimes mimic liver steatosis. An ultrasound evaluation of hepatic steatosis should therefore be performed keeping these limitations in mind; the results should be cautiously interpreted, and the procedure should not be routinely recommended as a sole diagnostic or monitoring tool for NAFLD in children.

### Ultrasound Elastography

Ultrasound elastography is now being increasingly used in clinical practice, in order to aid the diagnosis and management of diffuse liver disease. The leading role of this technique relies on the differentiation between simple steatosis and non-alcoholic steato-hepatitis, in which liver storage is associated with inflammation and fibrosis. Several ultrasound elastography techniques have been defined, including transient elastography, supersonic shear wave elastography, acoustic radiation force impulse elastography (ARFI), and real-time tissue elastography. Transient elastography is performed with pulse-echo ultrasound, and measures liver stiffness as a function of the extent of liver infiltration. It can detect liver cirrhosis with high accuracy, although the accuracy is decreased at lower fibrosis stages [9,10]. In a study of 246 NAFLD patients, using ultrasound elastography for the diagnosis of moderate fibrosis, bridging fibrosis and cirrhosis were found to be 0.84, 0.93 and 0.95, respectively [11].

**Figure 2:**
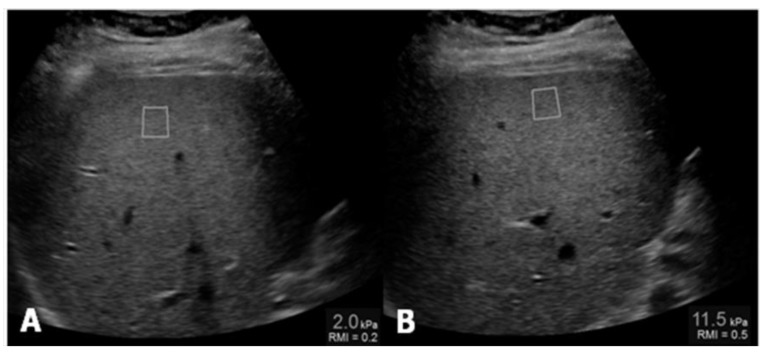
(**A**) Measurement of liver stiffness shows an estimated Young’s modulus of 2.0 kPa, below the cut-off for the diagnosis of stage F1 fibrosis; (**B**) Measurement of liver stiffness shows an estimated Young’s modulus of 11.5 kPa which reveals the presence of fibrosis.

Controlled attenuation parameter (CAP) has been proposed as a non-invasive method for the determination and measurement of hepatic steatosis. The mechanism of CAP is the reduction in amplitude of ultrasound that can be estimated as it is amplified through the liver tissue. This procedure uses the same radio-frequency data used for estimation of liver stiffness with Fibroscan (Echosens, Paris, France), an ultrasound-based vibration-controlled transient elastography device [12], comparing shear stiffness to that of a normal liver, which is between 6.5 and 7 kPa (Figure 2). 

In contrast, ARFI is used to examine the elasticity of a certain anatomical area during real-time B-mode imaging, with the help of a region-of-interest cursor. ARFI also performs similarly to CAP, but instead measures shearing velocity and compares it to the normal velocity of the liver (1 m/s), as this velocity is reduced when there is fatty infiltration [13].

## 3. Magnetic Resonance

**Figure 3:**
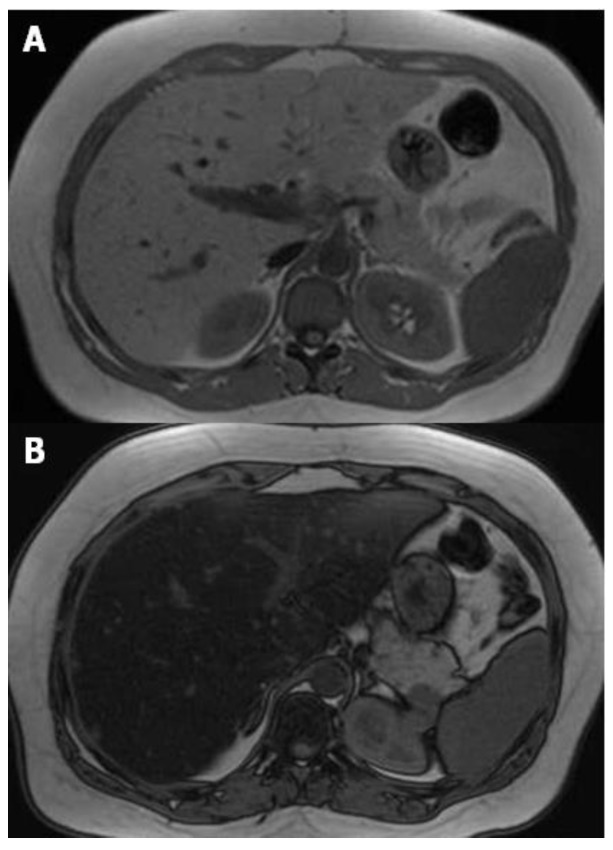
MR T1-weighted images with different echo times (TE) 4.2 (**A**) and 2.1 (**B**), show a marked drop of signal intensity in the out-of-phase image (**B**) which is a sign of severe steatosis.

Different methods can be performed for the evaluation of liver steatosis by magnetic resonance imaging (MRI), but the most widely preferred method is based on chemical shift imaging [14,15]. This technique is based on the different precession of protons linked to water and triglyceride molecules. Two sets of gradient-echo images of the liver are obtained, and echo-time-dependent signal interference between fat and water is considered. On in-phase echo-time, water and fat signals add up and therefore, the total signal intensity is higher. On out-of-phase echo-time, water and fat signals cancel out each other and consequently the total signal intensity decreases. Healthy liver has no difference in signal intensities between the in-phase and out-of-phase images, however, in the case of fat storage, the liver signal intensity diminishes on the out-of-phase image (Figure 3). This imaging method is reliable in the absence of magnetic field non-homogeneity and iron deposition. The main drawback is that the quantity of water and fat can affect their signals. This can be managed by acquiring new images with variable T1-weighting, through the application of two flip angles. High-flip-angle imaging is desirable for uncovering small amounts of fat in tissues that include mainly water, while low-flip-angle imaging should be applied for revealing small amounts of water in fat-rich tissues [16].

**Figure 4:**
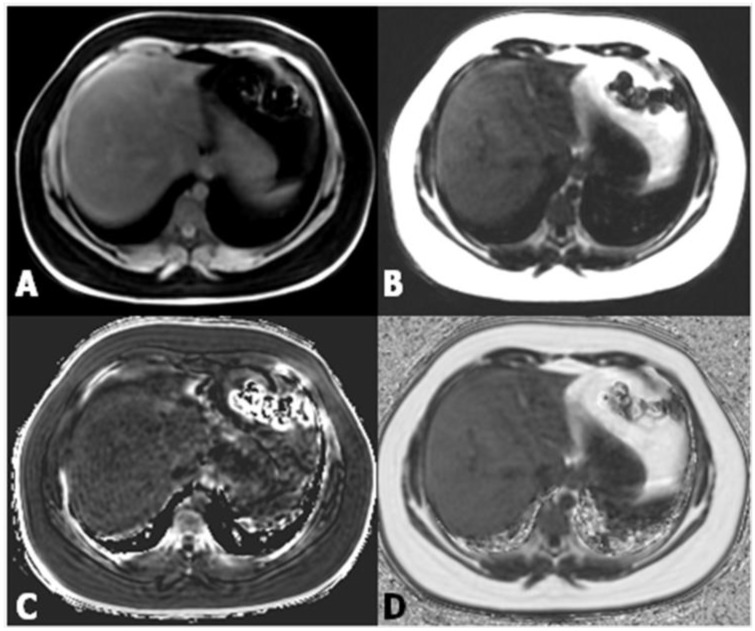
(**A,B**) Images from datasets acquired from a multi-echo, low flip angle sequence with clear separation of fat from nonfat tissue; (**C**) the R2 image is useful in the evaluation of iron concentration; (**D**) the fat-fraction image allows one to easily obtain the fat percentage by drawing a region of interest (ROI) within the parenchyma.

Proton density fat fraction (PDFF) measurement by MRI appears to be the most objective test for the quantification of liver steatosis, and has been recently adopted as a standard reference in clinical trials [17,18]. This technique overcomes the limits of dual-echo sequence, such as the T2* effect, T1 bias, spectral complexity of fat, eddy currents, noise bias, and magnetic non-homogeneity. PDFF is the ratio of density of mobile lipid protons (primarily triglycerides) and the total density of protons from water and fat. Moreover, it shows objective fat quantification and grading similar to magnetic resonance spectroscopy (MRS) in a single breath-hold, and measurement is easily performed by drawing a region of interest (ROI) on an automated PDFF map (Figure 4) [19].

Above all, it is very easy and practical for fat fraction measurement. In pediatric patients, comparison of biopsy and PDFF has shown promising results [20]. PDFF is an unconfounded and fundamental property of tissue, and it is insensitive to changes in acquisition parameters, thereby making it a robust quantitative biomarker [21]. PDFF is uniform across scanner platform and manufacturer and even field strength, and can standardize MRI-based fat quantification [22,23]. Moreover, simultaneous calculation of R2* and T2* maps from the same MRI sequence enables the estimation of hepatic iron content, which can be important for the diagnosis of co-existing fatty liver and hepatic iron overload [24,25].

**Figure 5:**
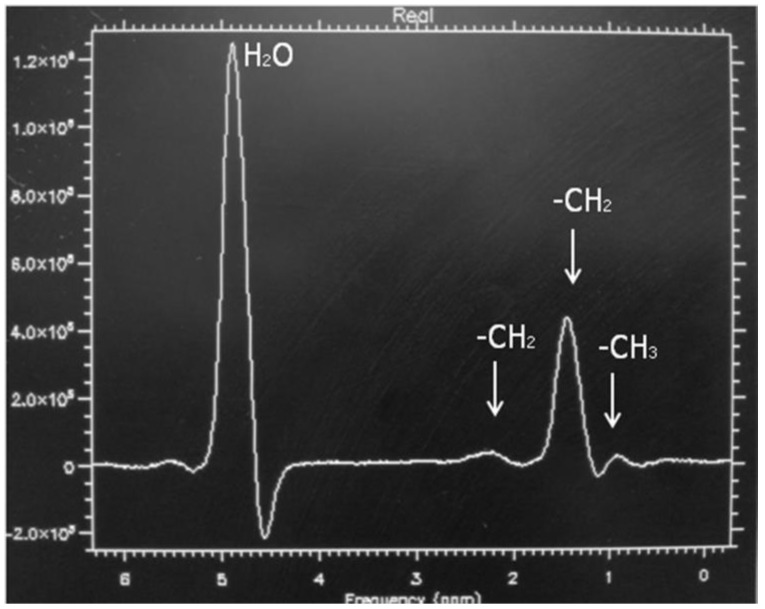
Magnetic resonance (MR) spectrum of a child with non-alcoholic fatty liver disease (NAFLD) and mild steatosis. Note the clear visualization of triglycerides peaks.

MRS is considered the non-invasive reference standard in the assessment of liver steatosis, because it is able to measure the real concentration of triglyceride within the hepatocytes in a specific volume, by analyzing protons of water and acyl-groups of the triglyceride differences in a specific resonance frequency domain [26,27,28,29]. The lipid (triglycerides) spectrum consists of multiple peaks that are identified at 0.9, 1.3, 2.0, 2.2, and 5.3 ppm, and whether the water peak is localized at 4.3 ppm. The dominant lipid peaks are caused by the resonance of methyl (–CH3) protons and methylene (–CH2–) in the triglyceride molecule (Figure 5) [30]. In normal cases, the dominant water peak is clear, and no triglyceride peaks are present. Existence of liver fat permits evaluation of a hepatic fat fraction via the area-under-water peak versus the area-under-fat peaks [31]. MRS has reported good correlation with histology for the evaluation of liver steatosis in children [32]. In the author’s experience, the ρ value has been measured at 0.68 and the sensitivity and specificity in the differentiation between healthy children from those with NAFLD, were 92.6 and 95.7, respectively [33].

However, MRS has some drawbacks, including that as a liver biopsy, it reflects only the lipid concentration in a small volume, so it does not depict the fat distribution along the liver parenchyma. Additionally, it requires a substantial amount of post-processing expertise, along with the fact that it is not routinely available on all scanners, so it may be impractical in some clinical settings.

### Magnetic Resonance Elastography

In the evaluation of patients with NAFLD, it is also important to establish the disease progression into non-alcoholic steato-hepatitis (NASH) and liver cirrhosis. Since NAFLD has the potential to progress into hepatic fibrosis, early diagnosis of fibrosis is important because, if treated, the degree of fibrosis can be minimized or reversed. Magnetic resonance elastography (MRE) can be used to quantify liver fibrosis, as this technique is based on the evaluation of the stiffness of liver parenchyma, and is a non-invasive technique [34]. It allows detection of fibrosis and differentiation of low-grade fibrosis from high-grade and it may also be feasible to distinguish steatosis from steato-hepatitis [35,36].

The MRE pulse sequences developed for adults have been modified for pediatric patients. This has been done by adopting the absorption rate and field of view for pediatric age groups, along with the power of the driver, which must be decreased by about 20% for patients between five and 18 years of age and by 40–50% for patients younger than two years, compared with the settings used in adult patients [37]. This power reduction allows for a reduction in abdominal pain. However, normal values of stiffness for pediatric age groups have not been suggested for MRE, because liver stiffness was found to not be related to age by the ultrasound-based transient elastography.

Importantly, the success rate and accuracy of MRE has been found to be higher than ultrasound-based transient elastography [38]. Furthermore, more of the liver can be evaluated with deep penetration, and ascites and obesity are not obstacles for examination. The main drawbacks of this technique are that it is expensive and it requires specific hardware and software, compared to conventional magnetic resonance equipment.

## 4. Conclusions

While ultrasound elastography is vital for its ability to clearly discriminate simple steatosis from NASH in the evaluation of the progression of NAFLD, among multiple imaging modalities, PDFF magnetic resonance accurately estimates fat storage within the liver parenchyma, is easy to use, and offers an overview of fat distribution within the organ. Therefore, this technique should be considered as the non-invasive reference standard for the diagnosis of liver steatosis. Further research studies should be focused on the non-invasive differentiation between simple steatosis and NASH considering serum-markers, imaging modalities and their association.

## References

[1] Ratziu V., Charlotte F., Heurtier A., Gombert S., Giral P., Bruckert E., Grimaldi A., Capron F., Poynard T. (2005). Sampling variability of liver biopsy in nonalcoholic fatty liver disease. Gastroenterology.

[2] Kotronen A., Yki-Jarvinen H. (2008). Fatty liver: A novel component of the metabolic syndrome. Arterioscler. Thromb. Vasc. Biol..

[3] Younossi Z.M., Gramlich T., Liu Y.C., Matteoni C., Petrelli M., Goldblum J., Rybicki L., McCullough A.J. (1998). Nonalcoholic fatty liver disease: Assessment of variability in pathologic interpretations. Mod. Pathol..

[4] Vajro P., Lenta S., Socha P., Dhawan A., McKiernan P., Baumann U., Durmaz O., Lacaille F., McLin V., Nobili V. (2012). Diagnosis of nonalcoholic fatty liver disease in children and adolescents: Position paper of the ESPGHAN Hepatology Committee. J. Pediatr. Gastroenterol. Nutr..

[5] Clemente M.G., Mandato C., Poeta M., Vajro P. (2016). Pediatric non-alcoholic fatty liver disease: Recent solutions, unresolved issues, and future research directions. World J. Gastroenterol..

[6] Strauss S., Gavish E., Gottlieb P., Katsnelson L. (2007). Interobserver and intraobserver variability in the sonographic assessment of fatty liver. AMR Am. J. Roentgenol..

[7] Cengiz M., Sentürk S., Cetin B., Bayrak A.H., Bilek S.U. (2014). Sonographic assessment of fatty liver: Intraobserver and interobserver variability. Int. J. Clin. Exp. Med..

[8] Awai H.I., Newton K.P., Sirlin C.B., Behling C., Schwimmer J.B. (2014). Evidence and recommendations for imaging liver fat in children, based on systematic review. Clin. Gastroenterol. Hepatol..

[9] Talwalkar J.A., Kurtz D.M., Schoenleber S.J., West C.P., Montori V.M. (2007). Ultrasound-based transient elastography for the detection of hepatic fibrosis: systematic review and meta-analysis. Clin. Gastroenterol. Hepatol..

[10] Friedrich-Rust M., Ong M.F., Martens S., Sarrazin C., Bojunga J., Zeuzem S., Herrmann E. (2008). Performance of transient elastography for the staging of liver fibrosis: A meta-analysis. Gastroenterology.

[11] Wong V.W., Vergniol J., Wong G.L., Foucher J., Chan H.L., Le Bail B., Choi P.C., Kowo M., Chan A.W., Merrouche W., Sung J.J., De Lédinghen V. (2010). Diagnosis of fibrosis and cirrhosis using liver stiffness measurement in nonalcoholic fatty liver disease. Hepatology.

[12] Polyzos S.A., Mantzoros C.S. (2014). Necessity for timely noninvasive diagnosis of nonalcoholic fatty liver disease. Metabolism.

[13] Yoneda M., Suzuki K., Kato S., Fujita K., Nozaki Y., Hosono K., Saito S., Nakajima A. (2010). Nonalcoholic fatty liver disease: US based acoustic radiation force impulse elastography. Radiology.

[14] Venkataraman S., Braga L., Semelka R.C. (2002). Imaging the fatty liver. Magn. Reson. Imaging. Clin. N. Am..

[15] Cassidy H.F., Yokoo T., Aganovic L., Hanna R.F., Bydder M., Middleton M.S., Hamilton G., Chavez A.D., Schwimmer J.B., Sirlin C.B. (2009). Fatty liver disease: MR imaging techniques for the detection and quantification of liver steatosis. Radiographics.

[16] Bydder M., Yokoo T., Hamilton G., Middleton M.S., Chavez A.D., Schwimmer J.B., Lavine J.E., Sirlin C.B. (2008). Relaxation effects in the quantification of fat using gradient echo imaging. Magn. Reson. Imaging.

[17] Loomba R., Sirlin C.B., Ang B., Bettencourt R., Jain R., Salotti J., Soaft L., Hooker J., Kono Y., Bhatt A. (2015). Ezetimibe for the treatment of nonalcoholic steatohepatitis: Assessment by novel magnetic resonance imaging and magnetic resonance elastography in a randomized trial (MOZART trial). Hepatology.

[18] Noureddin M., Lam J., Peterson M.R., Middleton M., Hamilton G., Le T.A., Bettencourt R., Changchien C., Brenner D.A., Sirlin C. (2013). Utility of magnetic resonance imaging versus histology for quantifying changes in liver fat in nonalcoholic fatty liver disease trials. Hepatology.

[19] Idilman I.S., Aniktar H., Idilman R., Kabacam G., Savas B., Elhan A., Celik A., Bahar K., Karcaaltincaba M. (2013). Hepatic steatosis: Quantification by proton density fat fraction with MR imaging versus liver biopsy. Radiology.

[20] Tang A., Tan J., Sun M., Hamilton G., Bydder M., Wolfson T., Gamst A.C., Middleton M., Brunt E.M., Loomba R. (2013). Nonalcoholic fatty liver disease: MR imaging of liver proton density fat fraction to assess hepatic steatosis. Radiology.

[21] Reeder S.B., Hu H.H., Sirlin C.B. (2012). Proton density fat-fraction: A standardized MR-based biomarker of tissue fat concentration. J. Magn. Reson. Imaging.

[22] Vasanawala S.S., Yu H., Shimakawa A., Jeng M., Brittain J.H. (2012). Estimation of liver T2* in transfusion-related iron overload in patients with weighted least squares T2* IDEAL. Magn. Reson. Med..

[23] Chabanova E., Bille D.S., Thisted E., Holm J.C., Thomsen H.S. (2013). (1)H MRS assessment of hepatic steatosis in overweight children and adolescents: Comparison between 3T and open 1T MR-systems. Abdom. Imaging.

[24] Özcan H.N., Oğuz B., Haliloğlu M., Orhan D., Karçaaltıncaba M. (2015). Imaging patterns of fatty liver in pediatric patients. Diagn. Interv. Radiol..

[25] Hernando D., Levin Y.S., Sirlin C.B., Reeder S.B. (2014). Quantification of liver iron with MRI: State of the art and remaining challenges. J. Magn. Reson. Imaging.

[26] Thomsen C., Becker U., Winkler K., Christoffersen P., Jensen M., Henriksen O. (1994). Quantification of liver fat using magnetic resonance spectroscopy. Magn. Reson. Imaging.

[27] Szczepaniak L.S., Babcock E.E., Schick F., Dobbins R.L., Garg A., Burns D.K., McGarry J.D., Stein D.T. (1999). Measurement of intracellular triglyceride stores by H spectroscopy: Validation in vivo. Am. J. Physiol..

[28] Johnson N.A., Walton D.W., Sachinwalla T., Thompson C.H., Smith K., Ruell P.A., Stannard S.R., George J. (2008). Noninvasive assessment of hepatic lipid composition: Advancing understanding and management of fatty liver disorders. Hepatology.

[29] Mehta S.R., Thomas E.L., Bell J.D., Johnston D.G., Taylor-Robinson S.D. (2008). Non-invasive means of measuring hepatic fat content. World J. Gastroenterol..

[30] Qayyun A. (2009). MR Spectroscopy of the Liver: Principles and Clinical Applications. Radiographics.

[31] Lindback S.M., Gabbert C., Johnson B.L., Smorodinsky E., Sirlin C.B., Garcia N., Pardee P.E., Kistler K.D., Schwimmer J.B. (2010). Pediatric nonalcoholic fatty liver disease: A comprehensive review. Adv. Pediatr..

[32] Schwimmer J.B., Middleton M.S., Behling C., Newton K.P., Awai H.I., Paiz M.N., Lam J., Hooker J.C., Hamilton G., Fontanesi J. (2015). Magnetic resonance imaging and liver histology as biomarkers of hepatic steatosis in children with nonalcoholic fatty liver disease. Hepatology.

[33] Di Martino M., Pacifico L., Bezzi M., Di Miscio R., Sacconi B., Chiesa C., Catalano C. (2016). Comparison of magnetic resonance spectroscopy, proton density fat fraction and histological analysis in the quantification of liver steatosis in children and adolescents. World J. Gastroenterol..

[34] Talwalkar J.A., Yin M., Fidler J.L., Sanderson S.O., Kamath P.S., Ehman R.L. (2008). Magnetic resonance imaging of hepatic fibrosis: Emerging clinical applications. Hepatology.

[35] Wang Y., Ganger D.R., Levitsky J., Sternick L.A., McCarthy R.J., Chen Z.E., Fasanati C.W., Bolster B., Shah S., Zuehlsdorff S. (2011). Assessment of chronic hepatitis and fibrosis: Comparison of MR elastography and diffusion-weighted imaging. AJR Am. J. Roentgenol..

[36] Chen J., Talwalkar J.A., Yin M., Glaser K.J., Sanderson S.O., Ehman R.L. (2011). Early detection of nonalcoholic steatohepatitis in patients with nonalcoholic fatty liver disease by using MR elastography. Radiology.

[37] Serai S.D., Towbin A.J., Podberesky D.J. (2012). Pediatric liver MR elastography. Dig. Dis. Sci..

[38] Towbin A.J., Serai S.D., Podberesky D.J. (2013). Magnetic resonance imaging of the pediatric liver: Imaging of steatosis, iron deposition and fibrosis. Magn. Reson. Clin. N. Am..

